# Prediction of severe erectile dysfunction after penile fracture repair: machine learning analysis results from the reconstruction and trauma working group of the society of urological surgery (RAT-SUS)

**DOI:** 10.1093/sexmed/qfaf101

**Published:** 2025-12-17

**Authors:** Serdar Geyik, Ismail Onder Yilmaz, Mehmet Zubaroglu, Mutlu Deger, Rahmi Kavak, Hilmi Sari, Yavuz Onur Danacıoglu, Çaglar Sertkaya, Mehmet Yilmaz, Ibrahim Haciobey, Mustafa Tıpırdamaz, Mehmet Dündar, Mesut Berkan Duran, Can Sinirsiz, Omer Bayrak, Onur Zeytun, Ali Can Albaz, Murat Demir, Yunus Emre Göger, Murat Ucar, Burak Akgül, Ersan Arda, Ilker Akarken, Ahmet Güzel, Mehmet Vehbi Kayra, Ibrahim Güven Kartal, Reha Girgin, Dursun Baba, Gökhan Ceker, Mehmet Özen, Ahmet Gürbüz, Ozgür Yilmaz, Ozan Bozkurt

**Affiliations:** Department of Urology, Aksaray Training and Research Hospital, 92nd Avenue No. 1, Tacin Neighborhood, Aksaray Merkez District, Aksaray 68200, Türkiye; Department of Urology, Çukurova University Balcalı Hospital, Güney Kampüs Boulevard 15/10, Balcalı Neighborhood, Sarıçam District, Adana 01330, Türkiye; Department of Urology, Çukurova University Balcalı Hospital, Güney Kampüs Boulevard 15/10, Balcalı Neighborhood, Sarıçam District, Adana 01330, Türkiye; Department of Urology, Çukurova University Balcalı Hospital, Güney Kampüs Boulevard 15/10, Balcalı Neighborhood, Sarıçam District, Adana 01330, Türkiye; Department of Software Engineering, Adana Alparslan Turkes Science and Technology University, Balcali Neighborhood, Catalan Street No. 201/1, Saricam District, Adana 01250, Turkey; Department of Urology, Ankara Etlik City Hospital, Halil Sezai Erkut Street No. 5, Varlık Neighborhood, Yenimahalle District, Ankara 06170, Türkiye; Department of Urology, Bakırköy Dr. Sadi Konuk Training and Research Hospital, Dr. Tevfik Sağlam Street No. 11, Zuhuratbaba Neighborhood, Bakırköy District, Istanbul 34147, Türkiye; Department of Urology, Bakırköy Dr. Sadi Konuk Training and Research Hospital, Dr. Tevfik Sağlam Street No. 11, Zuhuratbaba Neighborhood, Bakırköy District, Istanbul 34147, Türkiye; Department of Urology, Atlas University, Faculty of Medicine, Anadolu Street No. 40, Hamidiye Neighborhood, Kağıthane District, İstanbul 34408, Türkiye; Department of Urology, Başakşehir Çam and Sakura City Hospital, G-434 Street No. 2L, Başakşehir Neighborhood, Başakşehir District, İstanbul 34480, Turkey; Department of Urology, Aydın Adnan Menderes University, Faculty of Medicine, Central Campus, Zafer Neighborhood, 09100 Efeler, Aydın, Türkiye; Department of Urology, Aydın Adnan Menderes University, Faculty of Medicine, Central Campus, Zafer Neighborhood, 09100 Efeler, Aydın, Türkiye; Department of Urology, Pamukkale University Faculty of Medicine, Kınıklı Campus, 20070 Kınıklı, Pamukkale / Denizli, Turkey; Department of Urology, Dokuz Eylül University Faculty of Medicine, Balçova Campus, Mithatpaşa Street No. 1606, Balçova District, İzmir 35340, Turkey; Department of Urology, Gaziantep University Faculty of Medicine, Şehitkamil Campus, Üniversite Boulevard No. 12, Şehitkamil District, Gaziantep 27310, Turkey; Department of Urology, Gaziantep University Faculty of Medicine, Şehitkamil Campus, Üniversite Boulevard No. 12, Şehitkamil District, Gaziantep 27310, Turkey; Department of Urology, Manisa Celal Bayar University Faculty of Medicine, Üniversite Street, Hafsa Sultan Hospital Campus, Yunusemre District, Manisa 45030, Turkey; Department of Urology, Dursun Odabaş Medical Center, Van Yüzüncü Yıl University, Zeve Campus, Tuşba District, Van 65090, Turkey; Department of Urology, Meram Faculty of Medicine, Necmettin Erbakan University, Yunus Emre Neighborhood, Beysehir Street No. 281, Meram District, Konya 42090, Turkey; Department of Urology, Alanya Training and Research Hospital, Alanya Alaaddin Keykubat University, Oba Neighborhood, Hacikadiroglu Street No. 4, Alanya District, Antalya 07460, Turkey; Nevsehir State Hospital, 15 Temmuz Neighborhood, 148th Street No. 1, Nevsehir Merkez District, Nevsehir 50300, Turkey; Department of Urology, Trakya University Faculty of Medicine, Edirne 22100, Turkey; Department of Urology, Faculty of Medicine, Mugla Sitki Kocman University, Kötekli Campus, Mugla 48000, Turkey; Department of Urology, Aydin State Hospital, Hasan Efendi Neighborhood, 1901 Street No. 1, Efeler District, Aydin 09100, Turkey; Dadaloglu Neighborhood, 2591st Street No. 4, Yuregir District, Adana 01240, Turkey; Department of Urology, Kütahya Health Sciences University Evliya Çelebi Training and Research Hospital, Kütahya, Turkey; Department of Urology, Faculty of Medicine, Zonguldak Bülent Ecevit University, Zonguldak 67100, Turkey; Department of Urology, Faculty of Medicine, Düzce University, Konuralp Campus, Merkez / Düzce 81620, Turkey; Department of Urology, Başakşehir Çam and Sakura City Hospital, G-434 Street No. 2L, Başakşehir Neighborhood, Başakşehir District, İstanbul 34480, Turkey; Department of Urology, Samsun Training and Research Hospital, Kıran Neighborhood, 2164th Street No. 3, İlkadım District, Samsun 55090, Turkey; Nevsehir State Hospital, 15 Temmuz Neighborhood, 148th Street No. 1, Nevsehir Merkez District, Nevsehir 50300, Turkey; Department of Artificial Intelligence Engineering, Adana Alparslan Turkes Science and Technology University, Balcali Neighborhood, Catalan Street No. 201/1, Saricam District, Adana 01250, Turkey; Department of Urology, Dokuz Eylül University Faculty of Medicine, Balçova Campus, Mithatpaşa Street No. 1606, Balçova District, İzmir 35340, Turkey

**Keywords:** penile fracture, erectile dysfunction, machine learning, decision tree, postoperative complications

## Abstract

**Background:**

Erectile dysfunction (ED) is a significant complication following penile fracture repair, and early prediction is critical for clinical management.

**Aim:**

To evaluate the effectiveness of machine learning (ML) algorithms in predicting the development of severe ED after penile fracture repair and to identify complex risk factors beyond the scope of traditional statistical methods.

**Methods:**

A retrospective analysis was conducted using data from 547 patients who underwent surgical repair for penile fracture between January 2020 and June 2024 at 23 urology centers affiliated with the Reconstructive Urology and Trauma Study Group of the Urological Surgery Society. Patients were categorized into two groups based on their International Index of Erectile Function-5 scores at six months postoperatively: severe ED (+) (≤7) and ED (−) (>7). Eleven different ML classifiers were evaluated to determine the most predictive models. Four distinct resampling techniques were employed to address class imbalance in the dataset. Feature importance analysis was also performed to identify the most influential variables contributing to ED risk.

**Outcomes:**

This study was conducted to enable the early identification of patients at high risk of developing severe ED following penile fracture surgery.

**Results:**

Logistic Regression, Gaussian Naive Bayes, and Linear Support Vector Machine emerged as the best-performing algorithms on the original dataset, with Area Under the Curve (AUC) scores of 0.81, 0.78, and 0.76, respectively. On the Synthetic Minority Over-sampling Technique (SMOTE)-resampled dataset, Quadratic Discriminant Analysis (QDA) achieved an AUC of 0.85, while the Artificial Neural Network (ANN) reached an AUC of 0.84. On the SMOTE-resampled dataset, QDA achieved a ROC-AUC of 0.85 (95% CI: 0.75-0.93), whereas on the SMOTE–Tomek Link–resampled dataset, the ANN attained a ROC-AUC of 0.84 (95% CI: 0.71-0.94). The most critical predictors of severe ED were age, comorbidities, tunical tear length, and time to surgery. Urethral injuries were not significant contributors, as all were minor and managed conservatively without urethroplasty.

**Clinical Implications:**

Integration of ML-based prediction models into clinical workflows could support early risk stratification and individualized patient care, ultimately improving postoperative functional outcomes.

**Strengths and Limitations:**

This study benefits from a large, multicenter dataset and a comparative analysis of multiple ML algorithms. However, its retrospective nature and inter-center variability in data reporting may limit generalizability.

**Conclusion:**

ML algorithms are effective and reliable tools for predicting severe ED after penile fracture repair and may enhance personalized postoperative management. Eliminating class imbalance in the data with resampling techniques improves model performance.

## Introduction

Penile fracture is a rare urological emergency involving rupture of the tunica albuginea during erection (ED).^1^ In Europe, approximately 60% of cases occur during sexual intercourse,^2^ with manual manipulation and turning in bed also reported as common causes.^3^ Although surgical repair is standard, complications such as ED, penile curvature, and painful erections may occur.^4^ The incidence of ED after repair ranges from 1.9% to 13.9%.^2^

Several risk factors for post-fracture ED have been identified, including older age, tunical tear length, delayed surgery, smoking, bilateral rupture, and anxiety.^5-7^ However, no large-scale study has applied machine learning (ML) to predict severe ED following penile fracture repair. ML algorithms can uncover complex, non-linear patterns not easily detectable through conventional statistics.^8^^,^^9^ While widely used in uro-oncology, ML remains underutilized in reconstructive urology.^10^

This multicenter study aimed to develop a high-performance ML-based model to predict severe ED using the “Penile Fracture Database,” comprising clinical, surgical, and long-term follow-up data from 23 centers within the Reconstructive Urology and Trauma Study Group (RAT-SUS). The model, employing logistic regression, random forest, and decision tree algorithms, is expected to aid in early identification of high-risk patients and improve postoperative management.

## Materials and methods

This multicenter, retrospective cohort study was conducted using the “Penile Fracture Database,” which includes comprehensive data from 23 urology clinics affiliated with the Society of Urological Surgery – Reconstructive Urology and Trauma Study Group (RAT-SUS). Between January 2020 and June 2024, a total of 750 patients who underwent surgical repair for penile fracture were registered in the database. Patients with urethral injuries requiring urethral reconstruction, those lacking preoperative International Index of Erectile Function-5 (IIEF-5) scores, and those with incomplete data were excluded from the study. After applying these exclusion criteria, 547 patients were included in the final analysis. Data were retrieved from electronic medical records and encompassed a wide range of variables, including patient demographics, comorbidities, preoperative ED status, etiology of the fracture, time to surgery, fracture location, tunical tear length, presence of urethral injury, extent of corpus cavernosum damage, and type of surgical incision.

For cases with missing preoperative or six-month postoperative IIEF-5 data, medical records were reviewed in detail, and when necessary, patients were contacted by phone or interviewed during outpatient visits to complete the questionnaire. Although this approach was implemented to ensure data completeness, we acknowledge that reliance on patient self-reporting may introduce a risk of misclassification bias.

The length of the tunical tear was assessed based on intraoperative direct visualization and, when available, preoperative imaging findings. The length of the tunical tear was assessed based on intraoperative direct visualization and, when available, preoperative imaging findings.

All participating centers were members of the Society of Urological Surgery – Reconstructive Urology and Trauma Study Group (RAT-SUS). Prior to data collection, a standardized protocol and data collection template were developed and distributed to all centers. This form included clear definitions of all variables and measurement criteria to ensure consistency across sites.

The study group held regular meetings throughout the study period to monitor data quality, resolve discrepancies, and ensure data security and harmonization. All submitted data were reviewed by the principal investigator, and any incomplete, inconsistent, or inaccurate entries were excluded from the final analysis. This standardization process ensured inter-center reliability and data integrity.

Inclusion criteria comprised confirmation of penile fracture diagnosis by clinical examination or imaging methods, having undergone surgical repair, being 18 years of age or older, availability of at least six months of postoperative follow-up, and assessment of erectile function at six months postoperatively using the IIEF-5 questionnaire.

Exclusion criteria included patients with incomplete medical records, missing six-month follow-up data, absence of IIEF-5 scoring, concomitant genital trauma, or those lost to follow-up or with incomplete follow-up information.

ED was assessed using the IIEF-5 questionnaire at the six-month postoperative follow-up. In this study, the ML models were designed specifically to predict severe ED, defined as an IIEF-5 score ≤ 7. Patients with scores >7—including those with mild or moderate ED—were grouped as ED (–) and not modeled separately, as the primary aim was to identify clinically significant functional impairment.

Following the application of these inclusion and exclusion criteria, patients were categorized into two groups based on the development of severe ED after penile fracture repair. According to the IIEF-5 scores obtained at the six-month postoperative evaluation, patients with a score of ≤7 were classified as the severe ED (+) group, while those with a score > 7 were categorized as the severe ED (−) group.^11^

### ML analyses and model evaluation

This study assessed 11 ML algorithms to predict ED after penile fracture in 547 patients (37 with ED, 510 without). The dataset, including demographic, clinical, and laboratory variables, was split into training (70%) and test (30%) sets. Algorithms such as Logistic Regression, Support Vector Machine (SVMs), Random Forest, and Neural Networks were compared using 10-fold cross-validation. AUC-ROC was chosen as the main performance metric for its threshold-independent evaluation. Hyperparameters were tuned for selected models (eg, K-Nearest Neighbors [KNN] with 5 neighbors, Random Forest via RandomizedSearchCV, Linear SVM with C = 0.025, class_weight = “balanced”, RBF SVM with gamma = 1, and MLP with alpha = 1, max_iter = 1000). Others used default settings. Decision Tree optimization was performed using GridSearchCV, and the final model was built with the most relevant features.

There was a significant class imbalance in the original dataset, with the proportion of cases with severe ED being 6.8%. To address this imbalance, resampling techniques such as Synthetic Minority Over-sampling Technique (SMOTE), Adaptive Synthetic (ADASYN), Random Oversampling, and SMOTE–Tomek Link were applied exclusively during the training phase. For the SMOTE method, the minority class sample size was increased to half the size of the majority class, whereas in the other methods, the minority class was oversampled to achieve parity with the majority class sample size. For each resampling method, models were retrained, and evaluation was performed while preserving the integrity of the test set (the test set was never resampled at any stage). Table 1 presents a comparative summary of the class-wise sample sizes in the training dataset before and after the application of resampling techniques.

**Table 1:** Class distribution before and after applying different resampling techniques.

**Dataset**	**No Severe ED**	**Severe ED**
**Original (Unbalanced)**	93.24%	6.76%
**SMOTE**	66.67%	33.33%
**SMOTE–Tomek Link**	50.00%	50.00%
**ADASYN**	50.00%	50.00%
**Random Oversampling**	50.00%	50.00%

In the original unbalanced dataset, the Severe ED class constitutes only 6.76% of cases, reflecting a pronounced class imbalance that could bias model training toward the majority (No Severe ED) class. After applying resampling techniques: SMOTE increases the minority class proportion to 33.33%, partially reducing imbalance while retaining more majority-class samples. SMOTE–Tomek Link, ADASYN, and Random Oversampling all produce a perfectly balanced dataset (50%-50%), ensuring equal representation of both classes.

Performance metrics included ROC-AUC, Precision-Recall AUC (average precision), and Brier Score. Furthermore, 95% confidence intervals for the ROC-AUC were estimated using a bootstrap procedure with 2000 iterations.

To develop the ML models, Python programming language version 3.11 (Python Software Foundation, 2023)^12^ and PyCharm IDE version 2024.3 (JetBrains, 2023)^13^ were utilized. The implementation of ML algorithms was carried out using the Scikit-learn library version 1.6 (Pedregosa et al.).^14^

The technical specifications of the devices used in this study are as follows. The desktop computer is a DELL model equipped with an Intel Core i7-10 700 processor running at 2.90 GHz, 8 GB of RAM, a 500 GB SSD, and a 128 MB graphics card. The laptop is a Monster model featuring an Intel Raptor Lake Core i7-13700H processor, an NVIDIA GeForce RTX 4060 graphics card with 8 GB of GDDR6 memory, 32 GB of DDR4 RAM, and a 1 TB M.2 SATA NVME SSD.

### Surgical technique

All patients underwent emergency surgery under general or spinal anesthesia. Incision type depended on fracture location, with subcoronal or degloving approaches most commonly used. Hematomas were drained, and tunical tears were repaired using absorbable sutures. No major urethral injuries occurred; minor ones were managed conservatively. Flexible cystoscopy was performed when needed. Cavernosal injuries were repaired individually. All procedures followed standardized protocols, with at least six months of follow-up.

### Statistical analysis

Categorical variables were presented as frequencies and percentages, while numerical variables were summarized according to their distribution characteristics. Normally distributed continuous variables were expressed as mean ± standard deviation, whereas non-normally distributed variables were presented as median (minimum–maximum). The normality of continuous variables was assessed using the Shapiro–Wilk test. For comparisons between two independent groups, the Mann–Whitney U test was used for non-normally distributed data. The Chi-square test was employed to compare categorical variables between groups. All statistical analyses were performed using IBM SPSS Statistics for Windows, Version 25.0 (IBM Corp., Armonk, NY, USA). A *P*-value of less than .05 was considered statistically significant.

This study was conducted and reported in accordance with the Standards for Reporting Diagnostic Accuracy Studies guidelines for diagnostic accuracy research (Appendix 1).^15^

### Ethical approval and data confidentiality

This multicenter retrospective study was approved by the Aksaray University Non-Interventional Clinical Research Ethics Committee (Approval Date and Number: August 17, 2023 – 78-SBKAEK). The names of all 23 centers involved, members of the “Society of Urological Surgery – Reconstructive Urology and Trauma Study Group,” were submitted during the ethics application. In line with the Declaration of Helsinki and institutional guidelines, the requirement for informed consent was waived. No patient contact occurred, and all anonymized data were analyzed with strict adherence to confidentiality and data protection standards (Appendix 2).

## Results

In this study, demographic and clinical features were compared between patients with (*n* = 37) and without (*n* = 510) severe ED following penile fracture repair. Statistical analyses were performed using SPSS version 25. Since the data did not meet the assumption of normality (as assessed by Shapiro–Wilk test), continuous variables were expressed as median (min–max) and compared using the Mann–Whitney U test. Categorical variables were compared using the Chi-square test.

Patients with severe ED were significantly older than those without severe ED (59 [39-75] vs. 41 [12-83] years; *P* < .001). Additionally, the prevalence of severe ED was found to be higher among individuals with comorbidities, and this difference was statistically significant (*P* = .000) (Table 3).

Preoperative ED was significantly more frequent in the severe ED group compared to the non-ED group (37.8% vs. 7.6%; *P* < .001).

The most common etiology in both groups was sexual intercourse-related trauma (~59%; *P* = .813). The median time from injury to surgery was longer in the severe ED group (9 [1-144] vs. 7 [0.5-120] hours), although this was not statistically significant (*P* = .311).

Tunical tear length was significantly greater in patients with severe ED (15 [6-30] mm vs. 12 [1-50] mm; *P* = .026).

No significant differences were observed in urethral injury rates (10.8% vs. 11.2%; *P* = 1.000), corpus cavernosum injury pattern (*P* = .244), or incision type (*P* = .389) between the two groups.

**Figure 1:**
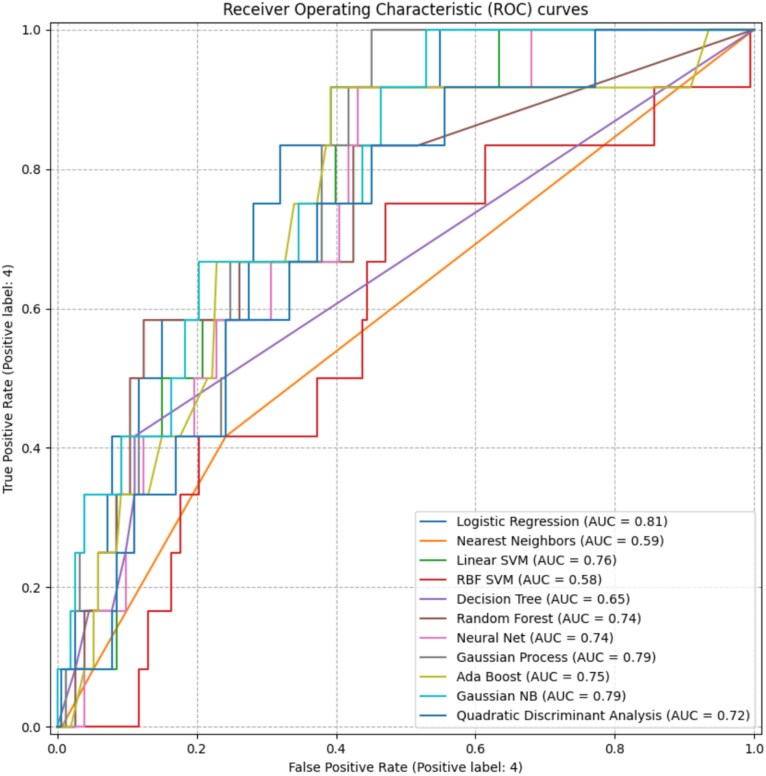
Presents the ROC curves of the eleven machine learning classifiers evaluated. The visual comparison highlights the variation in discriminative performance across models and complements the AUC-based results described above.

ML performance was evaluated using AUC metrics on the original dataset. Logistic Regression showed the best predictive performance (AUC = 0.81), followed by Linear SVM (AUC = 0.76) and Gaussian Naive Bayes. Tree-based models such as Random Forest and AdaBoost performed moderately but contributed to improved overall model accuracy. Overall, ML algorithms demonstrated strong potential in predicting ED risk after penile fracture, with Logistic Regression, Gaussian NB, and Linear SVM being the most effective classifiers (Figure 1).

**Figure 2:**
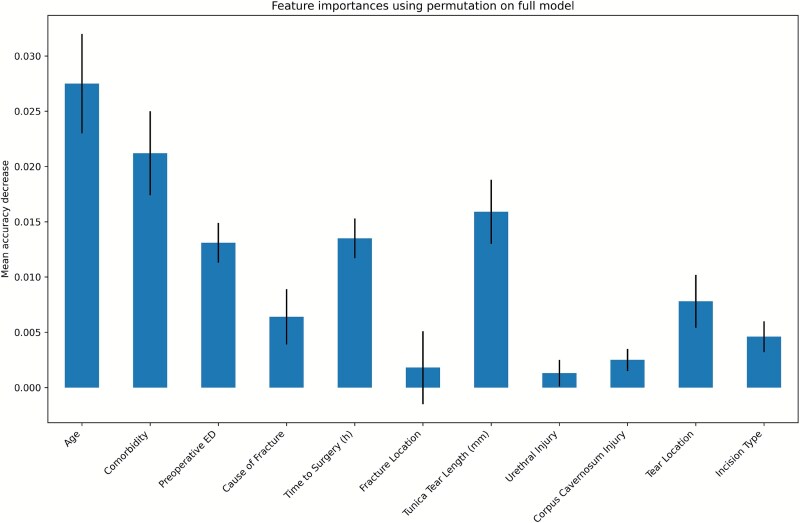
Displays a bar chart summarizing the permutation-based importance of each predictor in the final machine learning model. The visualization highlights the relative contribution of individual features to overall predictive performance.

In the original dataset, age, presence of comorbidities, preoperative ED, and tunical tear length emerged as the most influential predictors (Figure 2).

**Figure 3:**
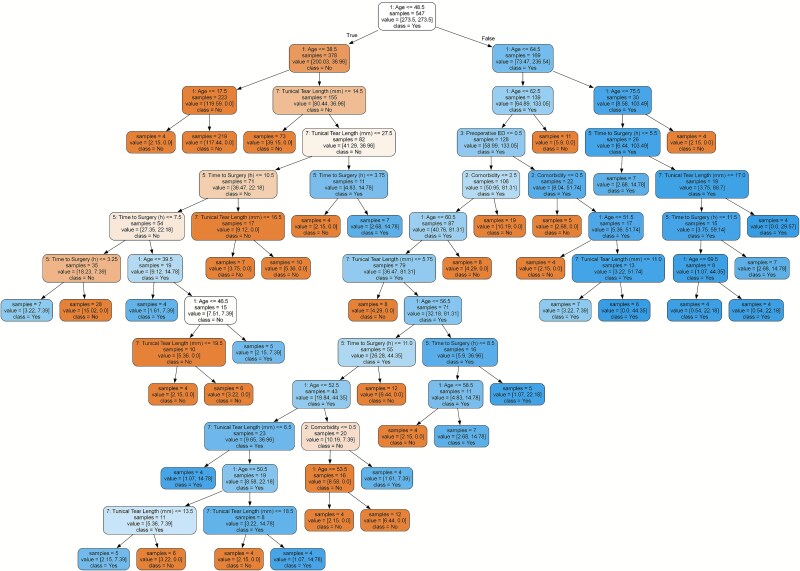
The visualization illustrates the key variables and corresponding threshold values employed by the decision tree during the classification process, thereby highlighting the model’s risk-based and interpretable decision pathways.

The decision tree model demonstrated a sensitivity of 0.27 and a specificity of 0.95. Figure 3 illustrates how key features—particularly age (≤46.9 years), tunical tear length (≤14.5 mm), and time to surgery (≤7.5 hours)—served as critical thresholds in risk stratification. The variable “age” appeared at multiple levels of the tree, with distinct split points such as ≤48.5 and ≤ 46.9 years, reflecting its layered contribution to classification. Comorbidities and preoperative ED further contributed to branching at lower tree levels.

**Figure 4:**
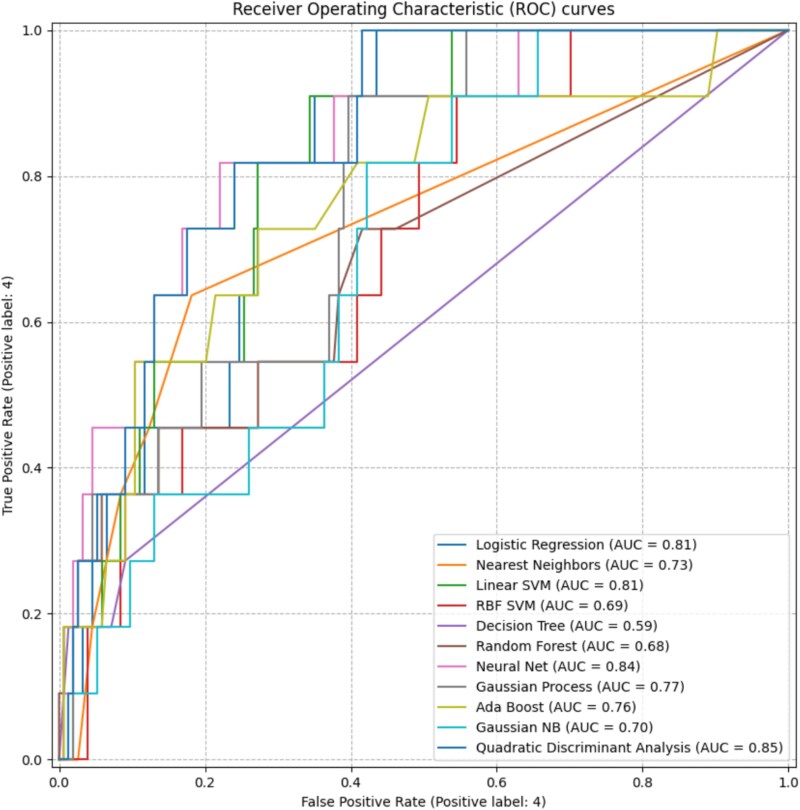
Compares the ROC curves of 11 machine learning models trained on the SMOTE-resampled dataset. The figure visually illustrates the variation in discriminative ability across models: QDA and Neural Net curves appear closer to the upper-left corner, reflecting stronger classification performance, whereas models such as Decision Tree and RBF SVM exhibit weaker separability between positive and negative classes.

The results indicate that balancing the datasets using resampling techniques generally improves model performance (Figure 4). Notably, the Quadratic Discriminant Analysis (QDA) model trained on the dataset balanced with the SMOTE method achieved the highest AUC of 0.85 (ROC-AUC: 0.85 [95% CI: 0.75-0.93]). This model also demonstrated remarkable performance with a precision-recall AUC value of 0.27. Furthermore, the Brier score, which measures the calibration of predicted probabilities against actual outcomes, was observed to be 0.0722.

Neural Network models, particularly when balanced using SMOTE–Tomek Link and ADASYN, achieved ROC-AUC values of 0.84, and low Brier scores (0.0628-0.0649). Although slightly lower in AUC compared to QDA, these models demonstrated competitive or higher PR-AUC values in some cases (eg, NN–SMOTE PR AUC: 0.32). Overall, the QDA and Neural Network models demonstrated the most substantial performance improvements following the application of resampling techniques. Comprehensive comparative results for all datasets are presented in Table 2.

**Table 2:** Best-performing models and evaluation metrics for original and resampled datasets.

**Dataset**	**Model**	**ROC-AUC (95% CI)**	**PR-AUC**	**Brier score**
Original (No resampling)	Logistic Regression	0.81 (0.71- 0.93)	0.1849	0.0981
SMOTE	QDA	0.85 (0.75- 0.93)	0.2705	0.0722
SMOTE–Tomek Link	Neural Net	0.84 (0.71- 0.94)	0.3073	0.0628
ADASYN	Neural Net	0.84 (0.71- 0.94)	0.2930	0.0649
Random oversampling	Neural Net	0.84 (0.72- 0.94)	0.2840	0.0676

Table 2 presents the best-performing model for each dataset configuration along with their evaluation metrics. In the original imbalanced dataset, Logistic Regression achieved a high ROC-AUC (0.81) but exhibited a relatively low PR-AUC (0.1849), indicating limited capability in identifying the minority class.

With SMOTE, QDA improved the ROC-AUC to 0.85 and achieved a notably higher PR-AUC (0.2705). Among the resampling approaches, SMOTE–Tomek Link combined with a Neural Network yielded the highest PR-AUC (0.3073) while maintaining a competitive ROC-AUC (0.84). ADASYN and Random Oversampling, both with Neural Networks, showed comparable ROC-AUC values (0.84) and slightly lower PR-AUC scores.

In terms of calibration, the lowest Brier Score (0.0628) was obtained with SMOTE–Tomek Link, suggesting improved probability estimation in the balanced setting. Overall, resampling techniques enhanced minority class detection and improved calibration compared to the original dataset.

**Figure 5:**
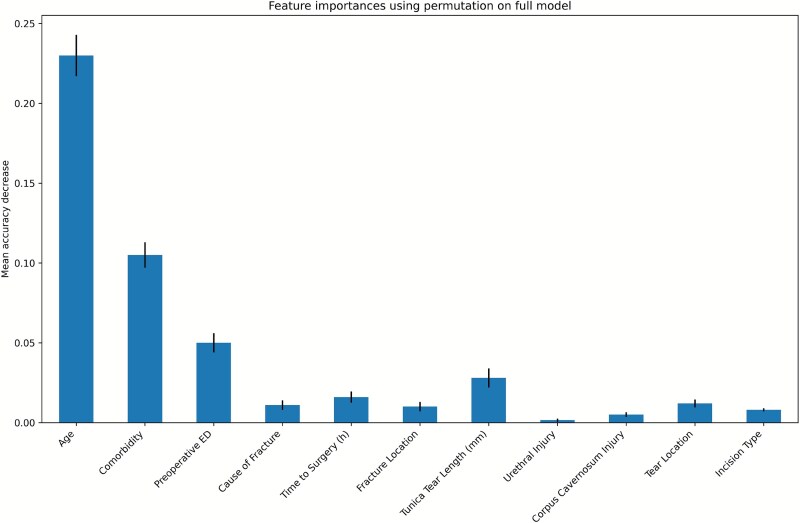
In the SMOTE-resampled dataset (Figure 5), Age and Comorbidity emerge as the most influential predictors, followed by Preoperative ED. Factors such as Tunical Tear Length, Time to Surgery, and Fracture Location show moderate impact, while others contribute minimally. These results suggest that demographic and clinical history variables have greater predictive power than anatomical or intraoperative factors.

In this study, the Random Forest algorithm was used to assess feature importance in predicting ED after penile fracture. In the original dataset, age emerged as the most influential variable, followed by comorbidities, tunical tear length, time to surgery, and preoperative ED. In the dataset resampled using the SMOTE technique, age, the presence of comorbidities, and preoperative ED were identified as the most influential variables, indicating their substantial contribution to predictive model performance (Figure 5).

These findings underscore the predictive value of specific clinical parameters and emphasize the importance of early intervention and comprehensive evaluation in reducing long-term ED risk. The decision tree provides a transparent framework for understanding the model’s logic based on hierarchical feature influence.

## Discussion

Previous studies in the literature have indicated that the development of severe ED following penile fracture is more commonly observed in older patients, with a particularly increased risk noted in men over the age of 45.^16-18^ Sharma et al. also reported that the risk of developing ED becomes more pronounced in patients over the age of 50.^17^ In our study, the median age was significantly higher in the group with severe ED compared to those without (*P* < .001, Table 3). This finding supports the notion that advanced age is a significant risk factor for the development of ED and is consistent with previous reports in the literature.

**Table 3:** Comparison of demographic, clinical, and surgical features between patients with and without severe erectile dysfunction following penile fracture.

**Features**		**Severe Erectyl Dysfunction (-)** **(*n* = 510)**	**Severe Erectyl Dysfunction (+)** **(*n* = 37)**	***P***
*Age, year*		41(12-83)	59(39-75)	***.000*** ^*^
*Comorbidities, % (n)*	No	79.2% (404)	40.5 (15)	***.000*** ^**^
	Diabetes mellitus	4,5% (23)	27 (10)	
	Cardiovascular disease	3.9% (20)	5.4 (2)	
	Others	9.6 % (49)	18.9 (7)	
	Diabetes mellitus + Cardiovascular disease	1.4 % (7)	5.4(2)	
	Diabetes mellitus + Others	0.4 (2)	2.8 (1)	
	Cardiovascular disease + Others	1 (5)	0 (0)	
*Preoperative ED, % (n)*	Yes	7.6 (39)	37.8 (14)	***.000*** ^**^
	No	92.4 (471)	62.2 (23)	
*Cause of Fracture, % (n)*	Bending while rigid	21.5 (110)	27 (10)	*.813* ^**^
	Manipulation during intercourse	58.6 (299)	59.5(22)	
	Masturbation	6.3 (32)	2.7 (1)	
	Others	13.4 (68)	10.8 (4)	
	Bending while rigid + Masturbation	0.2 (1)	0 (0)	
*Time to Surgery, Hour, (n)*		7 (0,5-120)	9 (1-144)	*.311* ^*^
*Fracture Localisation, % (n)*	Distal penile shaft	41.4 (209) 58.4 (284) 0.2(1)	35.1 (13) 64.9 (24) 0 (0)	*.527* ^**^
	Proximal penile shaft			
	Distal + proximal penile shaft			
*Tunical Tear Lenght, mm, (n)*		12 (1-50)	15 (6-30)	***.026*** ^*^
*Urethral Injury* ^***^ *, % (n)*	Yes	11.2 (57)	10.8 (4)	*1000* ^**^
	No	88.8 (453)	89.2 (33)	
*Corpus Cavernosum Injury, % (n)*	No	1.4 (7)	0 (0)	*.244* ^**^
	Unilateral	89.4 (456)	83.8 (31)	
	Bilateral	9.2 (47)	16.2 (6)	
*Incision Type, % (n)*	Fracture line	6.4 (49)	13.5 (5)	*.389* ^**^
	Circumcision	90.4 (461)	86.5 (32)	

^^*^^ *Normality was tested using SPSS, and the data were found to be non-parametric; therefore, the results were presented as median (min–max), and the Mann–Whitney U test was applied.*

^^**^^ *Normality was tested using SPSS, and the data were found to be non-parametric; therefore, the Chi-square test was used for the comparison of categorical variables.*

^^***^^ *Patients with urethral injuries not requiring urethral reconstruction.*

In individuals who developed severe ED following penile fracture, comorbidities such as diabetes mellitus and cardiovascular diseases were observed to be more prevalent. In a study conducted by Li et al.^19^ it was demonstrated that both the severity of ED and the time interval between the onset of ED and the diagnosis of coronary artery disease may serve as predictors of the severity of cardiovascular pathology. In a meta-analysis conducted by Zhao et al.^20^ ED was shown to significantly increase the risk of cardiovascular events, serving as an independent risk factor. In our study, diabetes mellitus and cardiovascular diseases were significantly more prevalent among patients who developed severe ED, indicating a higher burden of comorbidities in this group (*P* < .001, Table 3). These findings are statistically significant and consistent with the existing literature.

In previous studies, preoperative ED has not been identified as a specific risk factor for the development of ED following penile fracture. However, it is generally acknowledged that the presence of ED prior to the injury may influence postoperative sexual function outcomes. In a study by El-Assmy et al.^21^ it was noted that none of the patients had ED prior to the penile fracture, and the development of ED was attributed to the surgical intervention following the injury. Furthermore, the authors explicitly stated that there was no association between preoperative and postoperative ED. According to the findings of our study, severe ED more frequently observed in the group with a higher prevalence of preoperative ED (***P* < .001,**  Table 3). This observation represents a noteworthy finding within the context of the existing literature and may suggest a potential association that has not been clearly emphasized in previous studies.

In the literature, penile fracture is most commonly reported to occur during sexual intercourse, particularly in Western societies. In the study by Gürkan et al.^22^ 74.5% of penile fracture cases occurred during sexual intercourse, with the “doggy” and “man-on-top” positions being the most frequently reported mechanisms of injury. In our study, manipulation during sexual intercourse was identified as the most common cause of penile fracture in both groups, and this difference was not statistically significant (*P* = .813, Table 3). This finding is consistent with Western literature, which reports that penile fractures most frequently occur during sexual activity. The lack of a statistically significant difference suggests that the mechanism of injury alone may not be a decisive factor in the development of severe ED; rather, additional factors such as the severity of the injury, patient age, and comorbidities may collectively contribute to its occurrence.

Castro et al. specifically emphasized the strong association between tunical tears located in the proximal shaft and the development of postoperative ED. Similarly, Swanson et al. reported that penile fractures most frequently occur in the proximal shaft region.^23^ Although the proximal penile shaft was the most frequently involved fracture site in both groups, the difference between patients with and without severe ED was not statistically significant (*P* = .527, Table 3). This suggests that while proximal localization is commonly observed in penile fractures, it may not independently serve as a decisive factor in the development of severe ED.

Delayed surgical intervention may negatively impact the development of ED, whereas early surgical repair is often associated with better erectile function outcomes. Kati et al.^24^ reported that, in patients who sustained penile fractures due to direct trauma during sexual intercourse, early surgical repair was an effective approach in improving postoperative erectile outcomes. Ateyah et al.^25^ also stated that both early and delayed surgical repair can be effective in preserving postoperative erectile function in patients with penile fracture. In a study conducted by Kozacıoğlu et al.^26^ it was demonstrated that surgical repair of penile fracture within the first 24 hours did not lead to significant long-term deformity or ED. Furthermore, the authors noted that delaying surgical intervention for up to 24 hours had no adverse impact on long-term erectile function. In our study, the mean time to surgery was less than 24 hours in both groups; however, it was notably longer in the group with severe ED. Nevertheless, this difference was not statistically significant (*P* = .311, Table 3). While this finding may suggest a potential association between surgical delay and the development of severe ED, further large-scale prospective studies are needed to confirm this relationship.

Ortaç et al.^18^ found that larger tunical tears were significantly associated with higher rates of ED after penile fracture surgery. In a study conducted by Ragab et al.^27^ it was shown that each 1 cm increase in tunical tear length was associated with a 20.04-fold increase in the risk of developing ED. In our study, the tunical tear length was found to be significantly greater in patients who developed severe ED (*P* = .026, Table 3). This finding indicates that larger tunical defects constitute an important risk factor for the development of severe ED. Extensive tears in the tunica albuginea disrupt the structural integrity of the cavernosal tissue, impair the function of the veno-occlusive mechanism, and thereby directly contribute to the long-term development of persistent ED.

Barros et al. reported that the risk of developing ED is higher in penile fracture cases accompanied by urethral injury. Similarly, in a systematic review conducted by Shebl et al.^28^ the postoperative ED rate was reported to be 3.4% in cases of penile fracture with associated urethral injury, emphasizing that this complication is relatively rare. In our study, although the rate of urethral injury appeared slightly higher in the group without severe ED, the overall incidence of urethral injury remained low and comparable between both groups. This finding may be attributed to the absence of significant urethral injuries requiring direct urethroplasty among our patients, as well as the conservative management of cases with only minimal suspected urethral damage using supportive sutures. These observations suggest that carefully managed minimal urethral injuries may have a limited impact on postoperative erectile function. Therefore, it can be concluded that when addressed with meticulous surgical technique, minor urethral trauma does not appear to exert a significant negative effect on erectile outcomes.

In a study investigating the relationship between corporal involvement and ED following penile fracture, bilateral corporal involvement was identified as a factor that increases the risk of developing ED.^17^ Bilateral corpus cavernosum injuries were observed more frequently in the group that developed severe ED compared to the non-ED group; however, this difference was not statistically significant (*P* = .244, Table 3). Although this finding suggests a potential role of bilateral involvement, larger-scale studies are needed to establish a definitive association.

Hatzichristodoulou et al.^29^ emphasized that the type of surgical approach has a significant impact on postoperative erectile and voiding function. They specifically noted that the subcoronal incision is effective in reducing complication rates. Similarly, Castro et al.^30^ reported that the use of a subcoronal incision in penile fracture surgery reduces the risk of ED and penile curvature. In our study, the subcoronal incision was the most commonly preferred surgical approach in both groups—those with and without severe ED. Penoscrotal and longitudinal incisions were used less frequently. Although the distribution of incision types was similar between the groups, no significant association was observed between the type of incision and the development of severe ED.

ML, with its superior ability to analyze complex data structures and make predictions compared to traditional statistical methods, has been effectively applied in the healthcare sector—particularly in the context of big data and clinical decision support systems.

Studies conducted by Sharma et al.^31^ and Gürgen and Serttaş^32^ evaluated the effectiveness of ML methods in the diagnosis of heart diseases. Sharma et al. compared Random Forest, SVM, Naive Bayes, and Decision Tree models, reporting that Random Forest, Naive Bayes, and SVM achieved high accuracy and performed successfully, while the Decision Tree model showed relatively lower performance compared to the others. Similarly, Gürgen and Serttaş utilized KNN, Logistic Regression, Random Forest, and AdaBoost algorithms, and found that Random Forest achieved the highest predictive performance in terms of ROC-AUC.

As in other fields of healthcare, ML methods have become increasingly prevalent tools in urology for supporting clinical decision-making. Beyond oncological applications, ML has been effectively utilized in various urological subfields, including urolithiasis,^33^ infertility,^34^ urinary tract infections,^35^ and incontinence,^36^ demonstrating its versatility and potential in enhancing diagnostic accuracy and treatment planning across diverse clinical scenarios.

The prediction of pre- or postoperative complications represents a key application area for ML technologies in clinical practice. Given that the development of ED after penile fracture may be influenced by numerous factors, ML techniques offer a powerful means of analyzing these variables with greater accuracy and reliability. By integrating diverse biochemical and clinical data, ML models can assist in forecasting the risk of postoperative ED, thereby contributing to personalize treatment planning. These models can guide clinical decision-making by identifying high-risk patients early in the treatment process, facilitating timely intervention. Consequently, the postoperative recovery process can be more effectively monitored, and potential complications can be anticipated and managed more efficiently—ultimately improving patient outcomes and care quality.

​ In this study, the effectiveness of ML methods in predicting ED following penile fracture was evaluated, with a particular focus on the role of clinical and biopsy-related variables. While several ML applications in the literature have addressed the risk of ED after prostate biopsy, no dedicated study has been identified that specifically investigates the use of ML for predicting ED following penile fracture.

A comparative evaluation of all models and resampling strategies demonstrated that mitigating class imbalance substantially improved minority class detection performance. Overall, models trained with resampling techniques achieved higher ROC-AUC, Precision-Recall AUC (PR-AUC), and Brier scores compared to their counterparts trained on the imbalanced dataset (Table 2).

The findings show that in low-prevalence clinical datasets, SMOTE and related resampling techniques significantly increase class discrimination ability (ROC-AUC) and minority class detection performance (PR-AUC), while also improving the reliability of probability estimates (Brier score).The superior performance exhibited by QDA and Neural Network models on balanced datasets indicates the potential of resampling strategies to improve early detection success in clinical decision support systems. However, in certain models (eg, RBF SVM, Gaussian Process, Random Forest), sensitivity to the positive class remained near zero despite maintaining high specificity, suggesting that model decision boundaries remain sensitive even after addressing class imbalance.

Decision trees are powerful analytical tools that visualize complex decision-making processes and facilitate interpretability. These models aim to predict or classify a specific outcome by organizing a set of features or variables in a hierarchical structure. Each internal node represents a feature, the branches emanating from the node denote different values or ranges of that feature, and the leaf nodes provide the final prediction or classification. Interpreting decision trees involves understanding the decision thresholds at each level of the hierarchy and evaluating how these thresholds influence the outcome. This structure allows clinicians and researchers to trace the path of the model’s logic, making it easier to identify which variables play the most critical roles in the predictive process.

The root node of a decision tree contains the most influential discriminative variable in the model. The decision threshold at this node represents the value that best splits the dataset into two or more subgroups based on the target outcome. For instance, in the current model predicting ED following penile fracture, the root node variable is “Age”, with a threshold of “≤ 48.5”. This threshold serves as the initial branching point, indicating that patients older than 48.5 years fall into a higher-risk category for developing ED. As such, the decision threshold at the root node plays a pivotal role in shaping the overall predictive accuracy of the model, setting the foundation for subsequent decision paths within the tree.

The nodes and corresponding decision thresholds that follow the root node allow the model to define more specific subgroups. For example, among patients below a certain age threshold, the next most significant variable may be “Tunical tear length (mm)”, with a decision threshold of “≤ 27.5.” This threshold indicates that as the tunical tear length increases beyond 27.5 mm, the risk of developing ED also rises. These mid-level decision thresholds capture more nuanced distinctions within the dataset, thereby enhancing the model’s predictive capability. By progressively narrowing down the risk profile through such feature-based splits, the decision tree achieves a higher level of specificity in outcome prediction.

At the lower levels of the decision tree, additional variables such as “Time to surgery (hours)” and “Presence of comorbidities” further refine the risk stratification process through their respective decision thresholds. These thresholds enhance the model’s ability to assess postoperative ED risk with greater precision in specific patient subgroups. For instance, with regard to time to surgical intervention, the risk of developing ED increases sharply as the duration (in hours) before surgery becomes longer. This finding underscores the clinical importance of early intervention protocols. The leaf nodes at the bottom of the decision tree represent the final outcomes of all sequential decisions made by the model. At this level, patients are assigned to a specific risk category—such as “ED present” or “ED absent”—based on the cumulative influence of all preceding decision thresholds.

In conclusion, decision trees play a critical role in understanding the decision-making mechanism of predictive models and in identifying which factors are most influential for a given outcome. Much like in clinical decision-making processes, they serve as valuable tools for risk assessment and outcome prediction, particularly in postoperative patient monitoring across various surgical fields. Additionally, the presence of multiple split thresholds for the same variable (eg, age ≤ 48.5 and ≤ 46.9) within the decision tree does not indicate inconsistency. Instead, it reflects the algorithm’s ability to adaptively partition the dataset at different levels based on local feature interactions—a known and expected behavior in hierarchical models such as decision trees. Their transparent and interpretable structure allows clinicians to trace model logic, making them especially useful for guiding individualized treatment strategies and improving patient care.

This study aims to address a significant gap in the literature by utilizing ML techniques to predict the development of severe ED following penile fracture. The application of ML algorithms has enabled the identification of subtle interactions and patterns among clinical parameters that are often difficult to capture using traditional statistical methods. This data-driven approach not only improves predictive accuracy but also provides a robust foundation for personalized postoperative patient management. In particular, interpretable models such as decision trees offer clinicians actionable insights for early risk stratification. The findings of this study support the integration of ML-based tools into clinical workflows, and highlight the need for future prospective studies to validate these models across larger and more diverse patient populations.

The findings obtained in this study demonstrate that ML algorithms offer higher accuracy and reliability in predicting ED following penile fracture compared to traditional clinical assessment methods. Notably, the logistic regression model achieved the best performance with an AUC value of 0.81 on the test dataset, yielding results that are consistent with previously reported risk factors in the literature. Our analysis identified significant associations between ED development and key variables such as age, time to surgery, tunical tear length, and presence of comorbidities. These findings underscore the importance of personalized patient management and risk assessment for postoperative complications. Overall, the interpretation of these results is expected to assist clinicians in more effectively predicting outcomes for patients with penile fracture and in formulating optimal treatment strategies tailored to individual risk profiles.

In our study, conventional statistical analyses revealed statistically significant associations between severe ED and the variables of age, presence of comorbidities, preoperative ED status, and tunical tear length. These variables were shown to be clinically important independent risk factors for poor postoperative functional outcomes.

Beyond identifying individual predictors, we applied machine ML-based decision tree models, which enabled the delineation of precise threshold values for these key variables. Specifically, patients older than 48.5 years, with a tunical tear length exceeding 14.5 mm, and a time to surgery longer than 7.5 hours were positioned at critical branching points in the decision tree and were categorized as high-risk for severe ED. These thresholds lay the groundwork for a clinically applicable and easily interpretable risk classification system.

Notably, while “time to surgery” did not reach statistical significance in SPSS analysis, it emerged as a critical decision node in the ML model, further illustrating the strength of ML techniques in capturing complex, non-linear interactions that may not be evident through conventional analysis.

Together, these findings emphasize the value of integrating traditional statistics with ML approaches to enhance predictive accuracy, support individualized patient care, and ultimately improve long-term functional outcomes in patients undergoing surgical repair for penile fracture.

### Limitations

This study has several limitations. The retrospective design limits the ability to establish causal relationships and may introduce potential information bias. Since the data were collected retrospectively, the accuracy of patient responses—particularly in preoperative and postoperative assessments—may be compromised. Although missing data were supplemented through detailed review of medical records and direct patient contact, the reliance on self-reported IIEF-5 scores carries an inherent risk of misclassification bias.

The inclusion of 23 centers from various geographic regions introduces potential heterogeneity in surgical practices and data quality. Despite the implementation of a standardized data collection protocol and centralized data monitoring, variations in clinical practice and documentation procedures across centers cannot be entirely eliminated.

Moreover, the ML models were trained using the available variables and could not incorporate important clinical factors such as psychological status, frequency of sexual activity, and partner-related dynamics. The generalizability of the developed models also remains to be validated in external populations through future prospective multicenter studies.

## Conclusion

This multicenter study demonstrated that age, comorbidities, preoperative ED, and tunical tear length are significantly associated with the development of severe ED following penile fracture repair. Both conventional analyses and ML models supported these findings. Specifically, identified threshold values (age > 48.5 years, tunical tear >14.5 mm) offer clinically relevant guidance for risk stratification. These results contribute to the early identification of high-risk patients and support the implementation of personalized postoperative management strategies.

## Supplementary Material

Appendix_1_qfaf101

Appendix_2_qfaf101

## Data Availability

The datasets generated and/or analyzed during the current study are available from the corresponding author upon reasonable request.
