# Correction to: Prediction of severe erectile dysfunction after penile fracture repair: machine learning analysis results from the reconstruction and trauma working group of the society of urological surgery (RAT-SUS)

**DOI:** 10.1093/sexmed/qfag053

**Published:** 2026-06-25

This is a correction to: Serdar Geyik, Ismail Onder Yilmaz, Mehmet Zubaroglu, Mutlu Deger, Rahmi Kavak, Hilmi Sari, Yavuz Onur Danacioglu, Çaglar Sertkaya, Mehmet Yilmaz, Ibrahim Haciobey, Mustafa Tipirdamaz, Mehmet Dündar, Mesut Berkan Duran, Can Sinirsiz, Omer Bayrak, Onur Zeytun, Ali Can Albaz, Murat Demir, Yunus Emre Göger, Murat Ucar, Burak Akgül, Ersan Arda, Ilker Akarken, Ahmet Güzel, Mehmet Vehbi Kayra, Ibrahim Güven Kartal, Reha Girgin, Dursun Baba, Gökhan Ceker, Mehmet Özen, Ahmet Gürbüz, Ozgür Yilmaz, Ozan Bozkurt, Prediction of severe erectile dysfunction after penile fracture repair: machine learning analysis results from the reconstruction and trauma working group of the society of urological surgery (RAT-SUS), *Sexual Medicine*, Volume 13, Issue 6, December 2025, qfaf101, https://doi.org/10.1093/sexmed/qfaf101

In the originally published version of this manuscript, there was an error in the name of the seventeenth co-author within the author list and ‘Author contributions’ section.

This has been corrected to read: “Ali Can Albaz”.

