# Supplementary material for: Prediction of severe erectile dysfunction after penile fracture repair: machine learning analysis results from the reconstruction and trauma working group of the society of urological surgery (RAT-SUS)
Source: Sex Med. 2025 Dec 17;13(6):qfaf101. doi: 10.1093/sexmed/qfaf101 (PMC12710470; doi:10.1093/sexmed/qfaf101)
Supplement: Appendix_1_qfaf101 [file appendix_1_qfaf101.docx]

**APPENDİX 1(STARD_2015_CHECKLİST_)**

|  |  | Reporting Item | | Page Number/ explanation | | |
| --- | --- | --- | --- | --- | --- | --- |
| Title or abstract |  |  | |  | | |
| None | [#1](https://www.goodreports.org/reporting-checklists/stard/info/#1) | Identification as a study of diagnostic accuracy using at least one measure of accuracy (such as sensitivity, specificity, predictive values, or AUC) | | Title Page, Page 1 – Title includes “machine learning-based diagnostic accuracy model” and AUC is reported in Results section (p. 7) | | |
| Abstract |  |  | |  | | |
| None | [#2](https://www.goodreports.org/reporting-checklists/stard/info/#2) | Structured summary of study design, methods, results, and conclusions (for specific guidance, see STARD for Abstracts https://www.equator-network.org/reporting-guidelines/stard-abstracts/) | | Abstract, Page 1 – Structured abstract includes Background, Methods, Results, and Conclusion subsections | | |
|  |  |  |  |  |  |  |
| Introduction |  |  | |  | | |
| None | [#3](https://www.goodreports.org/reporting-checklists/stard/info/#3) | Scientific and clinical background, including the intended use and clinical role of the index test | | Penile fracture is a rare urological emergency, and severe ED may develop after surgical repair. The index test uses machine learning to predict this risk, supporting early risk stratification and personalized management | | |
| None | [#4](https://www.goodreports.org/reporting-checklists/stard/info/#4) | Study objectives and hypotheses | | To develop and compare machine learning models for predicting severe ED after penile fracture repair, hypothesizing superior predictive performance over traditional statistical methods. | | |
| Methods |  |  | |  | | |
| Study design | [#5](https://www.goodreports.org/reporting-checklists/stard/info/#5) | Whether data collection was planned before the index test and reference standard were performed (prospective study) or after (retrospective study) | | Retrospective study – Data were collected after the index test and reference standard were performed. |  |  |
|  |  |  |  |  |  |  |
| Participants | [#6](https://www.goodreports.org/reporting-checklists/stard/info/#6) | Eligibility criteria | | Adults (≥18 years) with clinically or radiologically confirmed penile fracture who underwent surgical repair, had ≥6 months postoperative follow-up, and completed IIEF-5 assessment at 6 months; exclusions were incomplete records, missing IIEF-5 data, concomitant genital trauma, or loss to follow-up | | |
| Participants | [#7](https://www.goodreports.org/reporting-checklists/stard/info/#7) | On what basis potentially eligible participants were identified (such as symptoms, results from previous tests, inclusion in registry) | | Potentially eligible participants were identified from the multicenter “Penile Fracture Database” registry, which included patients diagnosed with penile fracture based on clinical examination or imaging findings and treated surgically | | |
|  |  |  |  |  |  |  |
| Participants | [#8](https://www.goodreports.org/reporting-checklists/stard/info/#8) | Where and when potentially eligible participants were identified (setting, location and dates) | | Patients were identified between January 2020 and June 2024 at 23 urology centers affiliated with the Reconstructive Urology and Trauma Study Group of the Society of Urological Surgery (RAT-SUS). | | |
| Participants | [#9](https://www.goodreports.org/reporting-checklists/stard/info/#9) | Whether participants formed a consecutive, random or convenience series | | Participants formed a consecutive series of eligible patients meeting the inclusion criteria during the study period | | |
| Test methods | [#10](https://www.goodreports.org/reporting-checklists/stard/info/#10) | Index and reference tests in sufficient detail to allow replication | | Index test: Machine learning models using clinical and perioperative variables to predict severe ED (Methods, p. 7). Reference standard: IIEF-5 questionnaire at 6 months, severe ED defined as ≤7 (Methods, p. 8). | | |
| Test methods | [#11](https://www.goodreports.org/reporting-checklists/stard/info/#11) | Rationale for choosing the reference standard (if alternatives exist) | | IIEF-5 was chosen as it is a validated, widely used tool for assessing erectile function and quantifying ED severity (Methods, p. 8) | | |
| Test methods | [#12](https://www.goodreports.org/reporting-checklists/stard/info/#12) | Definition of and rationale for test positivity cut-offs or result categories of the index and reference tests, distinguishing pre-specified from exploratory | | Index test: Exploratory ML-derived cut-offs (p. 24). Reference standard: Severe ED pre-specified as IIEF-5 ≤ 7 (p. 8) | | |
|  |  |  |  |  |  |  |
| Test methods | [#13](https://www.goodreports.org/reporting-checklists/stard/info/#13) | Whether clinical information and reference standard results were available to the performers / readers of the index test; Whether clinical information and index test results were available to the assessors of the reference standard | | Clinical information was available to those performing the index test, but reference standard results were not (p. 7–8). Assessors of the reference standard had access to clinical information but not to index test results (p. 8) | | |
|  |  |  |  | | |  |
| Analysis | [#14](https://www.goodreports.org/reporting-checklists/stard/info/#14) | Methods for estimating or comparing measures of diagnostic accuracy | | ROC-AUC, Precision-Recall AUC, and Brier score were calculated; 95% CIs for ROC-AUC were obtained via 2,000-iteration bootstrap (p. 12) | | |
| Analysis | [#15](https://www.goodreports.org/reporting-checklists/stard/info/#15) | How indeterminate index test or reference standard results were handled | | Cases with incomplete or inconsistent data for either the index test or reference standard were excluded from analysis (p. 7–8) | | |
| Analysis | [#16](https://www.goodreports.org/reporting-checklists/stard/info/#16) | How missing data on the index test and reference standard were handled | | Missing index test or reference standard data were completed through medical record review and, if needed, patient contact; unresolved cases were excluded (p. 7–8) | | |
| Analysis | [#17](https://www.goodreports.org/reporting-checklists/stard/info/#17) | Any analyses of variability in diagnostic accuracy, distinguishing pre-specified from exploratory | | Exploratory analyses comparing diagnostic accuracy across different ML algorithms and resampling methods (Results, p. 21–24) | | |
| Analysis | [#18](https://www.goodreports.org/reporting-checklists/stard/info/#18) | Intended sample size and how it was determined | | All eligible patients meeting inclusion criteria with complete follow-up between January 2020 and June 2024 across 23 centers were included; no formal sample size calculation was performed (Methods, p. 7). | | |
| Results |  |  | |  | | |
| Participants | [#19](https://www.goodreports.org/reporting-checklists/stard/info/#19) | Flow of participants, using a diagram | | Not provided; from 750 registered cases, 547 included after exclusions (Methods, p. 7) | | |
| Participants | [#20](https://www.goodreports.org/reporting-checklists/stard/info/#20) | Baseline demographic and clinical characteristics of participants | | Table 3, p. 19 – Age, comorbidities, preoperative ED, etiology, time to surgery, tunical tear length, urethral injury, corpus cavernosum injury, incision type. | | |
| Participants | [#21](https://www.goodreports.org/reporting-checklists/stard/info/#21) | Distribution of severity of disease in those with the target condition, and distribution of alternative diagnoses in those without the target condition | | Not applicable (n/a) – Target condition defined as severe ED (IIEF-5 ≤7); non-ED group served as negative reference (Results, p. 19). | | |
| Participants | [#22](https://www.goodreports.org/reporting-checklists/stard/info/#22) | Time interval and any clinical interventions between index test and reference standard | | Not applicable (n/a) – Retrospective analysis; both index variables and reference standard were available at data collection (Methods, p. 7–8). | | |
| Test results | [#23](https://www.goodreports.org/reporting-checklists/stard/info/#23) | Cross tabulation of the index test results (or their distribution) by the results of the reference standard | | Not applicable (n/a) – No 2×2 cross-tabulation was produced; ML models generated probability scores, and diagnostic performance was reported with ROC-AUC, PR-AUC, and Brier scores (Results, p. 21–24). | | |
|  |  |  |  |  |  |  |
| Test results | [#24](https://www.goodreports.org/reporting-checklists/stard/info/#24) | Estimates of diagnostic accuracy and their precision (such as 95% confidence intervals) | | ROC-AUC, PR-AUC, F1-score, and Brier scores were reported; confidence intervals were provided using bootstrap methods (Results, p. 22–24). | | |
| Test results | [#25](https://www.goodreports.org/reporting-checklists/stard/info/#25) | Any adverse events from performing the index test or the reference standard | | n/a – As this was a retrospective data analysis, no adverse events related to the index or reference tests were reported (Methods, p. 7). | | |
| Discussion |  |  | |  | | |
| None | [#26](https://www.goodreports.org/reporting-checklists/stard/info/#26) | Study limitations, including sources of potential bias, statistical uncertainty, and generalisability | | The retrospective design, inter-center heterogeneity, limited subgroup sizes, and lack of an additional prospective validation cohort were discussed as limitations (Discussion, p. 28–29). | | |
| None | [#27](https://www.goodreports.org/reporting-checklists/stard/info/#27) | Implications for practice, including the intended use and clinical role of the index test | | Machine learning models may assist in predicting the risk of severe postoperative erectile dysfunction and have potential as clinical decision-support tools (Discussion, p. 30). | | |
| Other information |  |  | |  | | |
| None | [#28](https://www.goodreports.org/reporting-checklists/stard/info/#28) | Registration number and name of registry | | Study not registered | | |
| None | [#29](https://www.goodreports.org/reporting-checklists/stard/info/#29) | Where the full study protocol can be accessed | | not applicable (n/a) | | |
| None | [#30](https://www.goodreports.org/reporting-checklists/stard/info/#30) | Sources of funding and other support; role of funders | | not applicable (n/a) | | |

None The STARD checklist is distributed under the terms of the Creative Commons Attribution License CC-BY. This checklist can be completed online using <https://www.goodreports.org/>, a tool made by the [EQUATOR Network](https://www.equator-network.org) in collaboration with [Penelope.ai](https://www.penelope.ai)
