# Supplementary material for: Prediction of severe erectile dysfunction after penile fracture repair: machine learning analysis results from the reconstruction and trauma working group of the society of urological surgery (RAT-SUS)
Source: Sex Med. 2025 Dec 17;13(6):qfaf101. doi: 10.1093/sexmed/qfaf101 (PMC12710470; doi:10.1093/sexmed/qfaf101)
Supplement: Supplementary file 2 [file appendix_2_qfaf101.docx]

T.C

AKSARAY ÜNİVERSİTESİ

Klinik Araştırmalar Etik Kurul Başkanlığı

Tarih: 17/08/2023

Sayı: 78-SBKAEK

Konu: 2023/15-07 nolu karar

Sayın, Dr. Öğr. Üyesi Serdar Geyik

Aksaray Üniversitesi Aksaray Eğitim ve Araştırma Hastanesi

Üroloji

Tarafınızdan yürütülecek olan "Penil fraktür nedeniyle opere edilmiş hastalarda ameliyat sonrası 6. Ay ve veya daha sonrası periyotta, erektil fonksiyonun değerlendirilmesi ve erektil fonksiyon değerlendirme sonucu ile fraktür bölgesinin ilişkisinin olup olmadığının ortaya konulması" başlıklı proje 17.08.2023 tarihli toplantıda görüşülmüştür. Projeniz ile ilgili alınan karar ektedir.

Bilgilerinizi rica ederim.

Prof. Dr. Emine Arzu Köse

Klinik Araştırmalar Etik Kurulu Başkanı

T.C.
AKSARAY ÜNİVERSİTESİ
KLİNİK ARAŞTIRMALAR ETİK KURULU KARAR FORMU

ARAŞTIRMANIN AÇIK ADI	Penil fraktür nedeniyle opere edilmiş hastalarda ameliyat sonrası 6. Ay ve veya daha sonrası periyotta, erektil fonksiyonun değerlendirilmesi ve erektil fonksiyon değerlendirme sonucu ile fraktür bölgesinin ilişkisinin olup olmadığının ortaya konulması
VARSA ARAŞTIRMANIN PROTOKOL KODU	
ETİK KURUL PROTOKOL NUMARASI	78-SBKAEK

ETİK KURUL BİLGİLERİ	ETİK KURULUN ADI	Aksaray Üniversitesi Klinik Araştırmalar Etik Kurulu
	AÇIK ADRESİ:	Aksaray Üniversitesi Tıp Fakültesi Dekanlığı Bahçesaray Mh. Adana Yolu Üzeri 7 Km Kampüs 68100 AKSARAY
	TELEFON	0 382 288 2903
	FAKS	0 382 288 2999
	E-POSTA	tipetik@aksaray.edu.tr

BAŞVURU BİLGİLERİ	KOORDİNATÖR/SORUMLU ARAŞTIRMACI UNVANI/ADI/SOYADI	Dr. Öğr. Üyesi Serdar Geyik			
	KOORDİNATÖR/SORUMLU ARAŞTIRMACININ UZMANLIK ALANI	Üroloji			
	KOORDİNATÖR/SORUMLU ARAŞTIRMACININ BULUNDUĞU MERKEZ	Aksaray Üniversitesi Aksaray Eğitim ve Araştırma Hastanesi			
	VARSA İDARİ SORUMLU UNVANI/ADI/SOYADI				
	DESTEKLEYİCİ				
	PROJE YÜRÜTÜCÜSÜ UNVANI/ADI/SOYADI (TÜBİTAK vb. gibi kaynaklardan destek alanlar için)	Dr. Öğr. Üyesi Serdar Geyik			
	DESTEKLEYİCİNİN YASAL TEMSİLCİSİ	-			
	ARAŞTIRMANIN FAZI VE TÜRÜ	FAZ 1	☐		
		FAZ 2	☐		
		FAZ 3	☐		
		FAZ 4	☐		
		Gözlemsel ilaç çalışması	☐		
		Tıbbi cihaz klinik araştırması	☐		
		İn vitro tıbbi tanı cihazları ile yapılan performans değerlendirme çalışmaları	☐		
		İlaç dışı klinik araştırma	☐		
		Diğer ise belirtiniz X Retrospektif			
	ARAŞTIRMAYA KATILAN MERKEZLER	TEK MERKEZ X	ÇOK MERKEZLİ ☐	ULUSAL X	ULUSLARARASI ☐

	Belge Adı	Tarihi	Versiyon Numarası	Dili
DEĞERLENDİRİLEN BELGELER	ARAŞTIRMA PROTOKOLÜ	07.08.2023	V1	Türkçe X İngilizce ☐ Diğer ☐
	Bilgilendirilmiş Gönüllü Olur Formu-			Türkçe ☐ İngilizce ☐ Diğer ☐
	OLGU RAPOR FORMU			Türkçe ☐ İngilizce ☐ Diğer ☐
	ARAŞTIRMA BROŞÜRÜ			Türkçe ☐ İngilizce ☐ Diğer ☐

	Belge Adı		Açıklama
DEĞERLENDİRİLEN DİĞER BELGELER	SİGORTA	☐	
	ARAŞTIRMA BÜTÇESİ	☐	
	BİYOLOJİK MATERYEL TRANSFER FORMU	☐	

Etik Kurul Başkanının
Unvanı/Adı/Soyadı: Prof.Dr.E. Arzu Köse
İmza:

Not: Etik kurul başkanı, imzasının yer almadığı her sayfaya imza atmalıdır.

T.C.
AKSARAY ÜNİVERSİTESİ
KLİNİK ARAŞTIRMALAR ETİK KURULU KARAR FORMU

ARAŞTIRMANIN AÇIK ADI	Penil fraktür nedeniyle opere edilmiş hastalarda ameliyat sonrası 6. Ay ve veya daha sonrası periyotta, erektil fonksiyonun değerlendirilmesi ve erektil fonksiyon değerlendirme sonucu ile fraktür bölgesinin ilişkisinin olup olmadığının ortaya konulması
VARSA ARAŞTIRMANIN PROTOKOL KODU	
ETİK KURUL PROTOKOL NUMARASI	78-SBKAEK

	İLAN	☐	
	YILLIK BİLDİRİM	☐	
	SONUÇ RAPORU	☐	
	GÜVENLİLİK BİLDİRİMLERİ	☐	
	DİĞER:X	☐	1)Helsinki Bildirgesine uyulacağına dair taahhütnameler<br>2)Araştırmanın daha önce etik kurul değerlendirmesine sunulmadığına ve etik kurul onayı olmaksızın yapılmayacağına dair taahhütname<br>3)Literatürler<br>4)Araştırmacı özgeçmişi<br>5)Başvuru formu<br>6)Bütçe formu<br>7)Anabilim dalı izni<br>8)Başhekimlik izni<br>9)Etik kurul önerileri doğrultusunda yapılmış olan değişiklikler ve ilaveleri gösteren belgeler

**KARAR BİLGİLE Rİ**	**Karar No:2023/15-07**	**Tarih:17.08.2023**
	Yukarıda bilgileri verilen başvuru dosyası ile ilgili belgeler araştırmanın/çalışmanın gerekçe, amaç, yaklaşım ve yöntemleri dikkate alınarak incelenmiş ve uygun bulunmuş olup araştırmanın/çalışmanın başvuru dosyasında belirtilen merkezlerde gerçekleştirilmesinde etik ve bilimsel sakınca bulunmadığına toplantıya katılan etik kurul üyelerinin oy birliği ile karar verilmiştir.	

Etik Kurul Başkanının
Unvanı/Adı/Soyadı: Prof.Dr.E. Arzu Köse
İmza:

Not: Etik kurul başkanı, imzasının yer almadığı her sayfaya imza atmalıdır.

T.C.
AKSARAY ÜNİVERSİTESİ
KLİNİK ARAŞTIRMALAR ETİK KURULU KARAR FORMU

ARAŞTIRMANIN AÇIK ADI	Penil fraktür nedeniyle opere edilmiş hastalarda ameliyat sonrası 6. Ay ve veya daha sonrası periyotta, erektil fonksiyonun değerlendirilmesi ve erektil fonksiyon değerlendirme sonucu ile fraktür bölgesinin ilişkisinin olup olmadığının ortaya konulması
VARSA ARAŞTIRMANIN PROTOKOL KODU	
ETİK KURUL PROTOKOL NUMARASI	78-SBKAEK

KLİNİK ARAŞTIRMALAR ETİK KURULU

**ETİK KURULUN ÇALIŞMA ESASI**	İlaç ve Biyolojik Ürünlerin Klinik Araştırmaları Hakkında Yönetmelik, İyi Klinik Uygulamaları Kılavuzu
**BAŞKANIN UNVANI / ADI / SOYADI:**	Prof. Dr. E. Arzu Köse

**Unvanı/Adı/Soyadı**	**Uzmanlık Alanı**	**Kurumu**	**Cinsiyet**		**Araştırma ile ilişki**		**Katılım ***		**İmza**
			E	K	E	H	E	H	
Prof. Dr. E. Arzu Köse	Anesteziyoloji ve Reanimasyon	ASÜ Tıp Fakültesi Anesteziyoloji ve Reanimasyon ABD	☐	☒	☐	☐	☐	☐	
Prof. Dr. Aydın Rüstemoğlu	Tıbbi Biyoloji	ASÜ Tıp Fakültesi Tıbbi Biyoloji ABD	☒	☐	☐	☒	☒	☐	
Prof. Dr. Fadime Eroğlu	Tıbbi Parazitoloji	ASÜ Tıp Fakültesi Parazitoloji ABD	☐	☒	☐	☒	☒	☐	
Doç. Dr. Erdem Aras Sezgin	Ortopedi ve Travmatoloji	ASÜ Tıp Fakültesi Ortopedi ve Travmatoloji ABD	☒	☐	☐	☒	☒	☐	
Dr. Öğr. Üyesi Erdem Arslan	Tıbbi Farmakoloji	ASÜ Tıp Fakültesi Tıbbi Farmakoloji ABD	☒	☐	☐	☐	☐	☐	
Doç. Dr. Can Ateş	Biyoistatistk ve Tıp Bilişimi	ASÜ Tıp Fakültesi Biyoistatistik ve Tıp Bilişimi ABD	☒	☐	☐	☒	☒	☐	
Dr. Öğr. Üyesi Filiz Taşpınar	Fizyoloji	ASÜ Tıp Fakültesi Fizyoloji ABD.	☐	☒	☐	☒	☒	☐	
Dr. Öğr. Üyesi Melike Ordu	Patoloji	ASÜ Tıp Fakültesi Patoloji ABD	☐	☒	☐	☒	☒	☐	
Dr. Öğr. Üyesi A. Betül Öztürk	Çocuk Cerrahisi	ASÜ Tıp Fakültesi Çocuk Cerrahisi ABD	☐	☒	☐	☒	☒	☐	
Dr. Öğr. Üyesi Deniz Özkan	Radyoloji	ASÜ Tıp Fakültesi Radyoloji ABD	☐	☒	☐	☒	☒	☐	
Doç. Dr. Funda Çetinkaya	Hemşirelik	ASÜ Sağlık Bilimleri Fakültesi Cerrahi Hastalıkları Hemşireliği	☐	☒	☐	☐	☐	☐	
Dr. Öğr. Üyesi Cengizhan Kılıçaslan	Çocuk Sağlığı ve Hastalıkları	ASÜ Tıp Fakültesi Çocuk Sağlığı ve Hastalıkları ABD	☒	☐	☐	☐	☐	☐	
Çağrı Şavluğ	Avukat	Aksaray Şah Hukuk Bürosu	☒	☐	☐	☐	☐	☐	
Selçuk Kürşat Can	Eczacı	Selçuk Eczanesi	☒	☐	☐	☐	☐	☐	
Muhterem Öztürk	Sağlık Mesleği Mensubu Olmayan Üye	Serbest Meslek	☒	☐	☐	☐	☐	☐	

Etik Kurul Başkanının
Unvanı/Adı/Soyadı: Prof. Dr. E. Arzu Köse
İmza:

Not: Etik kurul başkanı, imzasının yer almadığı her sayfaya imza atmalıdır.
